# Contextual diversity during word learning through reading benefits generalisation of learned meanings to new contexts

**DOI:** 10.1177/17470218221126976

**Published:** 2022-10-25

**Authors:** Rebecca Norman, Rachael C Hulme, Christina Sarantopoulos, Varsha Chandran, Hantong Shen, Jennifer M Rodd, Holly Joseph, J. S. H Taylor

**Affiliations:** 1 Division of Psychology and Language Sciences, Department of Language and Cognition, University College London, London, UK; 2 Aston Institute of Health and Neurodevelopment and School of Psychology, Aston University, Birmingham, UK; 3 Institute of Education, University of Reading, Reading, UK

**Keywords:** Contextual diversity, word learning, reading, contextual variation, semantic diversity

## Abstract

From mid-childhood onwards, most new words are learned through reading. The precise meaning of many words depends upon the linguistic context in which they are encountered, which readers use to infer the appropriate interpretation. However, it is unclear what features of these linguistic contexts best support learning of new word meanings. We investigated whether learning words in contextually diverse sentences benefits word form and meaning learning in adults (*n* = 239). Participants learned meanings for 8 pseudowords through reading 10 sentences about each. Four pseudowords were learned in a diverse condition (10 sentences on different topics) and four were learned in a non-diverse condition (10 sentences on the same topic). An old-new decision post-test indicated that diversity did not influence word form learning. In a second post-test, participants chose which trained pseudoword completed a sentence from either an unfamiliar, untrained context, or a familiar, trained context. For familiar contexts, accuracy was higher for pseudowords learned in the non-diverse condition, but for unfamiliar contexts, accuracy was higher for pseudowords learned in the diverse condition. These results suggest that diverse contexts may promote development of flexible, decontextualised meaning representations that are easier to generalise to new contexts. Conversely, non-diverse contexts may favour extraction of context-bound representations that are more easily used in the same context.

## Introduction

Printed material is an invaluable language learning resource, containing low frequency words and complex syntactic structures rarely encountered in spoken language (Cunningham & Stanovich, 1998; Montag & MacDonald, 2015). Indeed, reading is fundamental in lifelong vocabulary acquisition, accounting for the majority of words acquired from mid-childhood onwards (Nagy et al., 1987). In addition, early reading skill predicts later declarative knowledge and language ability (Cunningham & Stanovich, 1997; Sparks et al., 2014). In order for a reader to successfully incorporate a new word encountered in print into their lexicon, they must extract both the word’s orthographic form (spelling) and meaning from the text (Perfetti & Hart, 2002). Word forms and meanings can be learned in as little as a single exposure under certain conditions (Coutanche & Thompson-Schill, 2014; Dollaghan, 1985). However, acquisition of word meaning knowledge is generally thought to be an incremental process (Frishkoff et al., 2011; Hulme et al., 2019) requiring readers to make inferences using linguistic information in the surrounding text (Joseph et al., 2014; Nagy & Gentner, 1990). For example, upon encountering the word *repeal* in the following sentence for the first time:

It was decided to **repeal** the additional tax on exports in 1966, and the current government seeks to abolish it completely.

We gain important clues to its meaning (“government,” “tax,” “abolish”). These linguistic cues also help readers to cope with the fact that the precise meanings of most words are dependent upon the contexts in which they occur. For example, *cup* can refer to either a container in “I broke my *cup*” or its contents in “may I borrow a *cup* of flour” (Li & Joanisse, 2021).

The “lexical quality hypothesis” (Perfetti & Hart, 2002) provides an explanation for how readers develop these flexible representations that allow the meaning of a word to vary somewhat in response to contextual cues. According to this theory, over repeated encounters readers develop a mental representation of a word that is both precise and flexible. Precision refers to a highly specified word form that can be clearly distinguished from other words. Flexibility allows words to be efficiently processed in a range of different situations. More specifically, words with high lexical quality are proposed to have context independent representations; that is their forms and meanings can be easily accessed without support from the surrounding linguistic context. On the other hand, for words with low lexical quality, readers must rely on contextual cues to infer word meanings. This means that such words are recognised slower when presented in isolation (Perfetti, 2007). Building on this, the “lexical legacy hypothesis” (Nation, 2017) suggests that differences in lexical quality emerge through experiencing a word in different linguistic environments over repeated encounters. Readers use new episodic and semantic information to learn aspects of a word’s meaning that would not be evident if the word was experienced in a uniform context. They also extract a word form representation that can be recognised regardless of the context in which it appears. Thus, the lexical legacy hypothesis predicts that words experienced in diverse linguistic contexts should have more flexible meaning representations that enable generalisation to new contexts, and more precise orthographic representations that facilitate form processing, as evidenced by faster and more accurate word recognition.

Initial support for the idea that contextual variation plays a role in lexical organisation comes from studies of lexical processing. Adelman et al. (2006) used the number of unique documents in which a word appears in a corpus as a metric of contextual diversity. They showed that this accounted for unique variance in lexical decision and word naming times over and above word frequency. This suggests that it is not just repetition that makes a word easier to process on future encounters, but also the diversity of the contexts in which these repetitions occur. However, Adelman et al.’s metric is insensitive to the semantic overlap between documents in which a word appears (Hoffman et al., 2013; Jones et al., 2012). For example, a word such as *tax* may occur in many documents, but which all relate to similar financial matters (Jones et al., 2012). This metric is, therefore, perhaps better described as “document count” rather than contextual diversity. Jones et al. (2012) created an alternative metric, termed semantic distinctiveness. This is calculated as the proportion of overlapping words across all documents in which a word occurs. For example, the word *perjury* is low in semantic distinctiveness as it only occurs in discussions of legal proceedings, whereas *predicament* is high in semantic distinctiveness as it can be used in a wide variety of contexts to describe a difficult dilemma (Hoffman et al., 2013). Jones et al. found that semantic distinctiveness accounted for unique variance in lexical decision and word naming responses over and above both word frequency and document count.

Hoffman and Woollams (2015) used a similar metric to investigate how contextual variation affects semantic as well lexical tasks. They termed this semantic diversity, which was calculated as the mean distance between all the contexts in which a given word occurred, using latent semantic analysis (LSA). Consistent with previous findings, lexical decision responses were faster and more accurate for high relative to low semantic diversity words. However, the reverse was true for a synonym judgement task. They suggested that experiencing words in diverse contexts leads to greater variability in semantic representations. This boosts initial semantic activation which facilitates lexical decision, but also creates less settled semantic patterns which impairs synonym judgement. However, synonym judgement does not test the core prediction of the lexical legacy hypothesis; that experiencing words in diverse linguistic contexts leads to better generalisation to new contexts.

One issue with corpus-based studies such as those described is that they only tell us that measures of contextual variability such as semantic distinctiveness and semantic diversity are correlated with lexical processing, not that they have a causal influence. Furthermore, these measures are highly correlated with other variables in natural language such as frequency, document count, and polysemy (Hoffman & Woollams, 2015; Jones et al., 2012), which are difficult to disentangle. Learning studies can help to address these issues by examining how varying levels of contextual diversity (i.e., the level of topic overlap) in training materials affects subsequent lexical processing. Furthermore, word learning studies allow for contextual diversity to be manipulated independently of other confounding variables, to establish how it affects both orthographic and semantic learning.

Johns et al. (2016) taught adults 10 pseudowords which were associated with the meanings of low-frequency English words (e.g., *constellation*). These were learned by reading five passages per word drawn from real-world sources. Five words were read in passages drawn from the same discourse topic (low diversity), and five were read in passages drawn from different topics (high diversity). The results were in line with Hoffman and Woollams (2015). High relative to low contextual diversity led to faster recognition of trained pseudowords in an old/new decision task, whereas low relative to high diversity trained pseudowords were rated as more similar to synonyms.

Mak et al. (2021) found a somewhat different pattern of results using a very similar paradigm. In Experiment 1, as in Johns et al. (2016), accuracy on a semantic relatedness task was higher for words learned in the low relative to the high diversity condition. However, old/new decision accuracy was also higher for low relative to high diversity words, although diversity did not influence reaction times (RTs). In Experiment 2, in which all words were repeatedly experienced in one discourse topic before diversity was introduced, the advantage for the low diversity condition on the semantic relatedness task was no longer present. Furthermore, old/new decision accuracy was higher and RTs were faster for words learned in the high relative to the low diversity condition. Mak et al. suggested that diversity may benefit word learning only once a stable representation has been established, which they termed “anchoring.” Taken together, the results of Johns et al. and Mak et al. support the idea that diversity facilitates word form recognition, but the effects on meaning learning are inconsistent. However, neither study examined whether experiencing words in diverse contexts leads to better generalisation of meaning, as predicted by the lexical legacy and lexical quality hypotheses.

Unlike Johns et al. (2016) and Mak et al. (2021), Bolger et al. (2008) found a diversity advantage for semantic judgements. Participants were taught the meanings of rare English words by reading four sentences. In the diverse condition, the sentences were designed to be contextually dissimilar using LSA. In the non-diverse condition, participants read the same sentence four times. Participants were more accurate at providing a definition or a synonym for diverse relative to non-diverse words. However, diversity had no impact on word recognition, assessed using an orthographic choice task. This may be because this task is perhaps more difficult than the lexical or old/new decision tasks used in other studies. They also investigated generalisation of meaning using a forced-choice sentence completion task that used a new context from those experienced in training. Diversity led to faster responses on this task, although accuracy did not differ between diverse and non-diverse items. This provides evidence that experiencing words in diverse sentence contexts facilitates semantic judgements that require a degree of generalisation, in line with the lexical legacy and lexical quality hypotheses.

Further evidence that diversity facilitates semantic judgements comes from Pagán and Nation (2019). As in Bolger et al. (2008), diversity was manipulated during training by presenting the to-be-learned word in the same sentence four times in the non-diverse condition and in four different sentences in the diverse condition. A two-alternative forced-choice comprehension question after training indicated that the meanings of the target words had been learned well (91% accuracy). Eye movement data collected during training revealed that words seen in diverse contexts were fixated on for longer than words experienced in repeated contexts. However, this pattern reversed in the post-exposure phase in which sentences were read in neutral sentences that did not provide cues to meaning. This suggests that diverse relative to repeated exposures led to better consolidation of knowledge about the learned words, allowing them to be more easily recognised and integrated into new contexts. However, these results may not truly reflect a benefit for word learning in diverse environments, but instead have resulted from attentional differences between the learning conditions. As the same sentence was repeatedly presented in the non-diverse condition, there were no additional cues to word meanings on subsequent presentations and attention may have declined over trials.

Joseph and Nation (2018) also tested generalisation of newly learned meanings but in children (mean age = 10.7 years) rather than adults. Participants learned meanings for six unfamiliar English verbs by reading a series of short sentences, which either shared a common context, for example, law (non-diverse condition), or were drawn from different contexts, for example, law, medicine, and finance (diverse condition). Each word was seen 10 times in either the diverse or non-diverse condition. Learning of word meanings was assessed using a cloze task in which children completed a sentence by selecting the correct learned word, and a plausibility task, in which they decided whether a sentence containing one of the learned words made sense. Importantly, some trials used a new context that had not been experienced during learning, thus requiring use of decontextualised knowledge of the target words. However, although performance was above chance on both tasks, there was no effect of diversity. Moreover, there was no effect of diversity on a spelling task used to assess word form learning. These null results differ from previous studies. It may be that children are less able to take advantage of variations in context than adults when learning new words and that repetition is more important (Hsiao & Nation, 2018).

To summarise, learning studies investigating the effect of contextual diversity on word learning have produced mixed results. Whereas some have found an advantage in word recognition (Johns et al., 2016; Mak et al., 2021 [Experiment 2]), only one found an advantage for meaning learning (Bolger et al., 2008). Others still have found no effect (Joseph & Nation, 2018), and some have reported a word recognition advantage for words learned in low diversity conditions (Mak et al., 2021 [Experiment 1]). Most importantly, no adult studies have yet examined whether contextual diversity during learning facilitates the formation of decontextualised meanings that can be generalised to new contexts.

### The present study

The primary aims of this study were twofold. First, to test the impact of learning words in diverse versus non-diverse linguistic environments on both lexical and semantic decisions. Second, to test the core prediction of the lexical legacy hypothesis; that encountering words in diverse linguistic contexts leads to better semantic processing when participants need to generalise to new contexts. To that end, we conducted a word learning study with adult participants and adapted the experimental design and materials from Joseph and Nation (2018) as they included semantic outcome measures that explicitly probed generalisation.

We operationalised diversity by comparing learning of words experienced in one discourse topic (non-diverse condition) versus multiple discourse topics (diverse condition), as in Johns et al. (2016) and Mak et al. (2021), rather than simply repeating a sentence for the non-diverse condition (Bolger et al., 2008; Pagán & Nation, 2019). This reflects how diversity is defined in the corpus-based literature and ensured that we were comparing learning in diverse versus non-diverse conditions rather than simply comparing the effects of meaning variation versus repetition (frequency). Although Joseph and Nation (2018+) described their manipulation as semantic diversity, we use the term contextual diversity to describe our manipulation as this more accurately reflects the fact that the to-be-learned words occurred in varying topics, but their core meanings did not vary. Joseph and Nation’s sentences were created for the purpose of their study, avoiding complex vocabulary found in real-world materials (e.g., Johns et al.’s passages focused on relatively obscure topics), which could have distracted from new word learning.

We made some methodological alterations. First, we replaced Joseph and Nation’s (2018) low-frequency target words with pronounceable pseudowords to ensure that adult participants did not have prior familiarity with the to-be-learned items. Second, we replaced the spelling test with an old/new decision task to test form recognition in a way that was directly comparable to previous studies with adults. Third, we did not include the plausibility task, since meaning learning and generalisation were assessed with the cloze task. Fourth, participants were instructed to learn the meanings of the new words and were informed that they would be tested on their knowledge of them later. Finally, diversity was manipulated within rather than between participants to increase power.

In line with the corpus-based lexical processing literature, we hypothesised that responses would be faster and more accurate on the old/new decision task for words learned in diverse relative to non-diverse contexts. We also predicted that responses would be faster and more accurate for words seen in the learning phase (trained items) than for untrained stimuli (foil items) in line with typical lexical decision tasks.

With respect to word meaning learning, in line with the lexical legacy hypothesis we predicted that there would be an interaction between contextual diversity at learning (diverse vs non-diverse) and the context of the cloze sentences (new vs old). Specifically, we predicted that for cloze sentences drawn from new contexts accuracy would be higher for items learned in the diverse relative to the non-diverse condition. Conversely, for cloze sentences drawn from a familiar context we expected accuracy to be higher for items learned in the non-diverse relative to the diverse condition.

## Methods

### Ethics

Ethical approval was granted by the University College London Language and Cognition Department Ethics Chairs, Project ID: LCD-2020-02.

### Participants

For practical reasons related to the availability of financial resources for payment of participants the data were collected in two phases associated with two separate student projects. There were some minor changes across these two experiments, and we thus include Experiment as a factor in the analysis. Specifically, one pseudoword differed between the two experiments (see Table 1), and they also included different additional post-tests that took place after those reported here. These were included for exploratory analyses as part of the separate projects and will not be discussed further. The data were analysed separately as part of these projects with analyses of variance (ANOVAs) but the LME analyses were only conducted after the full dataset was collected.

**Table 1.** Pseudowords and corresponding foils used in the two experiments.

Pseudoword	Foil	Experiment
invilled	invilted	1/2
lindered	lundered	1/2
sottled	sittled	1/2
danested	danepted	1/2
uzided	uzibed	1/2
noffled	naffled	1/2
perphised	perprised	1/2
tactorded	tactorned	1
rudgerbed	rudgerded	2

A total of 276 adults (83 in Experiment 1, 193 in Experiment 2)^1^ participated in this study. Participants were recruited online using Prolific (www.prolific.co) and were paid for their time (£7.50/hour). All were native English speakers with normal or corrected-to-normal vision and none reported a history of any developmental disorders or hearing impairments. Fifteen participants were excluded for reporting that they had taken notes (four from Experiment 1, 11 from Experiment 2), 12 participants were excluded for performing at or below chance (four or fewer correct answers out of eight) on a series of comprehension questions during the learning phase (three from Experiment 1, nine from Experiment 2). A further eight participants were excluded from Experiment 1 for having a native language other than English, and two additional participants were excluded from Experiment 2 for having previously participated in Experiment 1. All analyses reported here, unless otherwise specified, are based on the 239 remaining participants (142 females, mean age = 28.1 years, *SD* = 6.10).

### Design

Diversity was manipulated within-participants: in the learning phase participants saw four pseudowords embedded in non-diverse sentence frames, and four in diverse sentence frames, with each pseudoword being read in 10 different sentences. Cloze type was also manipulated within-participants: all participants were tested with one sentence from a familiar context and one from an unfamiliar context for each item in the cloze task. To account for any effects of some pseudowords or meanings being easier to learn than others, the assignment of pseudowords to diversity conditions as well as the assignment of pseudowords to meanings was counterbalanced across participants. Thus, we employed a 2x2 within-participants counterbalanced design, creating four different versions of the experiment for both Experiment 1 and Experiment 2 (see Table S1 and S2 available at https://osf.io/5xqrm/). Assignment of participants to each of the counterbalanced versions was randomised by the experimental software.

### Materials

#### Trained and foil pseudowords

In Joseph and Nation’s (2018) original study children learned the meanings of six unfamiliar real words (*Accumulated, Amalgamated, Exacerbated, Intervened, Confabulated, Languished*). To increase the power of our study, participants learned the meanings of two additional words (*Divulged, Thwarted*)^2^. For this adult study, we replaced the original targets with pseudowords that were phonotactically legal in English. The pseudoword targets, in addition to eight foil stimuli (which differed from each target pseudoword by a single letter) were taken from a word learning study by Hulme et al. (2021 July 29). They selected their items from materials developed by Mousikou et al. (2017), and Hulme et al.’s pretest showed that the foil and target stimuli were equivalent in terms of word likeness. As Joseph and Nation’s target words were all past tense verbs, all pseudowords for the current study were selected on the basis that they could be converted into plausible English past tense verbs by adding the -ed suffix (Ulicheva et al., 2020). The two sets of pseudowords differed by one item between Experiments 1 and 2 (see Table 1). This was due to an additional experimental manipulation in Experiment 2 to allow for an exploratory analysis of how ease of pronunciation affects word learning, which is not examined here.

#### Training sentences

Each to-be-learned pseudoword was embedded in 2 sets of 10 sentence frames, one set was diverse and one non-diverse. Joseph and Nation (2018) created these such that the non-diverse sentence frames all belonged the same context, whereas each sentence frame in the diverse condition belonged to a different context (see Table 2 for an example). To confirm the validity of the diversity manipulation, a separate group of adults rated how similar in topic the diverse and non-diverse sentences for each target word were to one another. Non-diverse sentences were rated as significantly more similar to one another than diverse sentences (Joseph & Nation, 2018). Sentences in the diverse and non-diverse conditions were matched in terms of length, *M* diverse = 145.88 vs. *M* non-diverse = 138.00; *t*(7) = 1.56, *p* = .163, and readability as indexed by the Flesch Reading Ease test, *M* diverse = 54.28 vs. *M* non-diverse = 55.34; *t*(7) = .43, *p* = .681. The sets of sentence frames were identical across both experiments.

**Table 2.** Example sentence stimuli for the original target word *accumulated*, here replaced by the pseudoword *invilled*. Note that the first sentence is the same in the diverse and the non-diverse conditions. All experimental sentence stimuli are provided in Table S3 available at https://osf.io/xbyjr/.

Non-diverse condition—shared context (Law/Evidence)	Diverse condition—different contexts
Enough proof had invilled so that the jury could make a fair judgement on the case.	Enough proof had invilled so that the jury could make a fair judgement on the case.
The police invilled a lot of strong evidence which meant they could arrest the thief.	The woman forgot to clean under the bed, so dust had invilled on the floorboards.
Members of MI5 invilled all the incoming data and saved it onto a computer file.	The girl loved collecting rubbers and invilled more each week using her pocket money.
After the news report went out, the police invilled more than 25 witnesses.	After just one week at his new school, the boy had already invilled several new friends.
The lawyer invilled witness statements to get support for the case.	The doctors invilled enough test results to diagnose and treat the patient.
The burglar invilled information about the neighbourhood before committing the crime.	Lava had invilled beneath the surface which caused a spectacular eruption from the volcano.
The evidence invilled until there was no question that he was guilty.	His debts invilled until he had to sell his house to pay off the loan.
The proof that she had stolen the money invilled over time and eventually she lost her job.	Although she had invilled a lot of wealth, this meant she also had to pay a lot of tax.
The witness statements invilled and in the end he decided to plead guilty.	She was shocked to discover how many emails had invilled while she was away.
The solicitor invilled the documents for the case and took them to court.	The fluid had invilled in his lungs and he found it very hard to breathe.

#### Cloze task sentences

For each pseudoword there were two sentences to complete, one “new” sentence, and one “old” sentence. New sentences were created from a new, unfamiliar context not seen for either diverse or non-diverse items during training. Old sentences used the same context as the non-diverse condition in the learning phase, which was also experienced in one learning trial for that item in the diverse condition (see Table 2). The cloze sentences in the original experiment used various tenses, which required participants to conjugate the verbs they had learned. We removed this requirement by converting all cloze sentences into the past tense, meaning that participants could use the learned pseudowords to complete the sentences without alteration. A full list of the cloze task sentences is provided in Table S4 available at https://osf.io/baqkp/.

### Procedure

**Figure 1.**
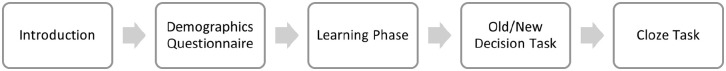
Outline of the experimental procedure.

The experiment comprised two phases within a single session: a learning phase and a test phase. Variations in contextual diversity were introduced in the learning phase. The test phase immediately followed and included two tasks: an old/new decision task, which measured learning of word forms, and a cloze task, which assessed learning of word meanings and participants’ ability to generalise these to new contexts. See Figure 1 for an overview of the experimental procedure.

All tasks were programmed and run using Gorilla (www.gorilla.sc) an internet-based platform. Participants were asked to complete the experiment in a quiet environment with no noise or other distractions to maximise their ability to concentrate.

#### Learning phase

Participants read a total of 80 unique sentence stimuli which were divided into 10 blocks. Each pseudoword appeared once in each block and sentence presentation order was randomised within blocks. Block presentation order was also randomised. One sentence for each of the to-be-learned pseudowords was followed by a comprehension question to encourage participants to read for understanding. The mean score in response to these questions was 86% indicating that participants were reliably reading for meaning. There were eight of these questions in total, meaning that 8 of the 10 blocks contained a comprehension question. These questions were the same in each version of the experiment and related to the content of the sentence, not to the meaning of the pseudoword, and were answered true or false. Participants received feedback on their responses. A full list of the comprehension questions and the corresponding sentence stimuli is provided in Table S5 available at https://osf.io/7u4dh/.

Participants were told that they would read a series of short sentences describing the meanings of new words. They were instructed to read these sentences silently in their heads and to learn the meanings of the new words from the information provided. They were informed that they would be tested on the new words and their meanings later in the study and asked not to take notes. Once the task began, each sentence was presented in the centre of the screen for a maximum of 12.5 s whereupon the next trial would begin automatically. Alternatively, participants could press “next” to advance to the next trial after reading the sentence. Each trial was followed by a 500 ms fixation cross. Participants had an optional 1-minute break after completing Blocks 3 and 6.

#### Test phase

Immediately following the learning phase, participants completed the old/new decision task followed by the cloze task. This ensured that the results of the old/new task could only be attributed to exposure during the learning phase, and not to additional familiarity with the word forms provided by the cloze task.

#### Old/new decision task

Participants were told that they would be presented with a series of words, some of which had been learned in the previous task and others that were spelled incorrectly. They were instructed to press the “j” key when they thought the word on screen was spelled the same as one they had seen in the previous task, and to press the “f” key when they thought it was spelled incorrectly, and to respond as quickly and as accurately as possible.

Each trial began with a 500 ms fixation cross after which the pseudoword appeared in the centre of screen. This remained on the screen until the participant entered a response. A blank screen was then presented for 100 ms before the next trial began automatically. In total participants completed 16 judgements, 8 for the target pseudowords and 8 for the foils. Presentation of the target pseudowords and foils was randomised. Accuracy and RT were recorded. No feedback was given.

#### Cloze task

Participants were told that they would see a series of sentences with one word missing, which would be one of the words they learned earlier. They were instructed to complete the sentences by selecting the correct word and were told that there was more than one sentence that goes with each word.

The task began with two practice trials. Participants were asked to complete a sentence presented at the top of the screen by selecting the correct answer from three options. There was no time limit on the trials. The missing word was a regular English past tense verb, which did not appear in either the sentence final or initial position, thus replicating the structure of the experimental trials. Participants were provided with feedback on their performance. After the practice trials, participants were reminded of the instructions before beginning the main task. The experimental trials consisted of a sentence presented at the top of the screen with a word missing corresponding to one of the learned pseudowords. All eight of the pseudowords seen in the learning phase were presented below the sentence, and participants had to select the one they thought completed the sentence. Participants saw 16 sentences in total, two for each pseudoword, and presentation order of the sentences was randomised. There was no time limit, and the next trial began as soon as the participant’s response was recorded. No feedback was given.

### Analysis plan

We set-out our analysis plan following data collection, but prior to carrying out the linear mixed-effects models analysis of the data. This analysis plan can be retrieved from: https://osf.io/asn8c/. Any deviations from this analysis plan have been noted.

#### Analytic approach

All data were analysed with linear mixed-effects models using the *lme4* package (version 1.1.26; Bates et al., 2015) in *R* (version 3.6.3; R Core Team, 2020). Generalised linear mixed-effects models were used for the old/new accuracy and cloze task data, while the old/new RT data were analysed using linear mixed-effects models. Contrasts were defined using deviation coding for diversity (diverse: 0.5 vs. non-diverse: -0.5) cloze type (new: 0.5 vs. old: -0.5), and Experiment (Experiment 1: 0.5 vs. Experiment 2: -0.5) with interactions coded by multiplying the contrasts for the relevant factors.

Specific details of the models are provided in the results section. Our approach to determining the final model random effects structure followed the procedure specified by Barr et al. (2013). In the first instance, models were computed with a maximal random effects structure: i.e., containing random intercepts for participant and item, and by-participant and by-item slopes for all factors of experimental interest^3^. If this model failed to converge or produced a singular fit, the random effects structure was simplified as follows: first, we removed the correlations between random intercepts and random slopes. If this model again failed to converge or produced a singular fit, we then removed the random intercepts from the model leaving the slopes intact. Should the model still fail to converge, we employed a forward model selection procedure starting with the simplest model (random intercepts only) and adding in the random slopes one at a time. Any models from this selection process that converged without producing a singular fit warning were compared to the simplest model using likelihood ratio tests. If none of the individual slope models provided a significantly better fit to the data (as indicated by a threshold of < .2 (Barr et al., 2013; Matuschek et al., 2017); then the intercepts only model was used as the final model. If any of the individual slope models were a significant improvement on the intercepts only model, the model with the lowest *p* value was compared against models with this slope and any other slope that converged individually. This procedure was repeated, taking the model with the lowest *p* value in each case until there was no significant improvement. For the generalised linear mixed-effects models for old/new accuracy and cloze task data the BOBYQA [Bound Optimization BY Quadratic Approximation] optimizer was used to facilitate model convergence (Bates et al., 2021). Significance of the fixed effects was determined by comparing the final model to a model with the fixed effect/interaction of interest removed using likelihood ratio tests. Full analysis scripts can be found at https://osf.io/z7fhq/.

### Results

#### Old/new decision task

**Figure 2.**
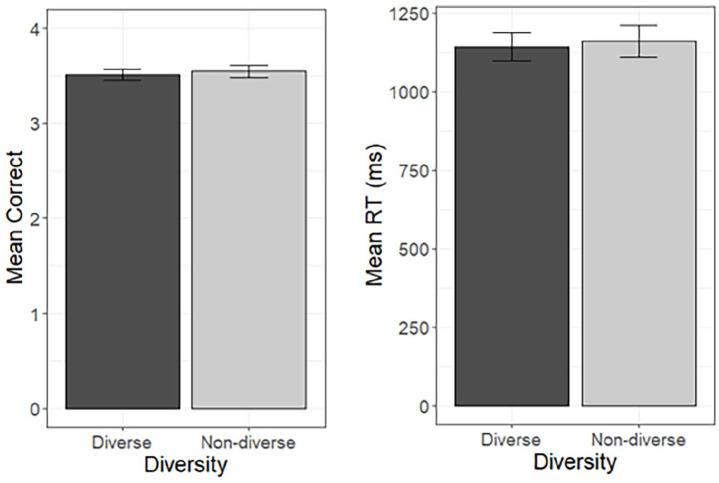
Mean number of correct responses (max = 4) and mean RT for correct responses in the diverse and non-diverse conditions in the old/new decision task. Error bars denote the standard error of the mean adjusted for the within participant design.

**Figure 3.**
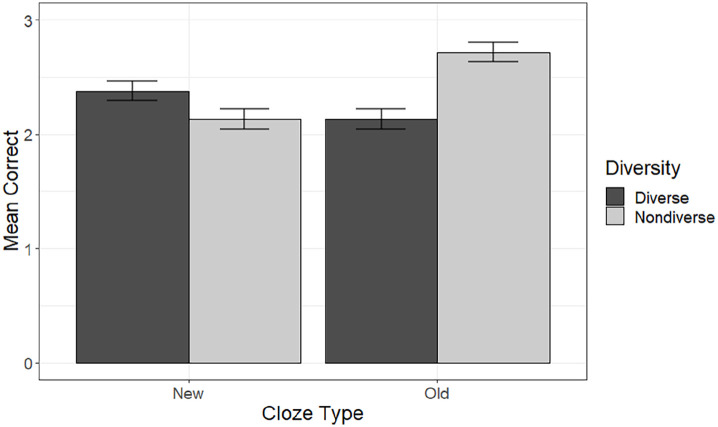
Mean number of correct responses (max = 4) on the cloze task in the diverse and non-diverse conditions for each cloze type. Error bars denote the standard error of the mean adjusted for the within participant design.

Overall accuracy was above chance (12 or more correct as identified using a binomial test) with participants making an average of 13.23/16 correct responses (*SD* = 3.13). For the analysis of RTs, only correct responses were analysed. Outliers were identified by visually inspecting histograms of the RT data. This resulted in any RTs above 9000 ms and below 300 ms being removed. Inspection of a histogram of the residuals and a scatterplot of the residuals vs. fitted values showed that assumptions of normality and homoscedasticity were violated, so log and inverse (1000/raw RT) transformations were applied. The inverse transformed RTs met the assumptions of normality and homoscedasticity most closely and were used for the final analyses. However, Figures 2 and 3 show untransformed RTs for ease of interpretation.

##### Trained versus foil items

We first checked the validity of the old/new task as a test of lexical decision by comparing accuracy and RTs on trained pseudoword stimuli and untrained foil trials. Three participants performed at floor (0/16 correct) meaning that the RT analysis was based on 236 participants. We expected it to be easier for participants to recognise trained items than to reject foil items, as evidenced by higher accuracy and faster RTs for trained items. A model with the maximal random effects structure was used for both analyses. This model contained stimulus type (trained vs. foil) as a fixed effect of interest. Fixed effects of Experiment (1 vs. 2) and the Experiment by stimulus type interaction were also included as control factors.

As expected, participants were significantly more accurate, χ^2^(1) = 20.19, *p* < .001, for trained items (*M* = 7.05, *SD* = 1.68) than foils (*M* = 6.18, *SD* = 1.86), and responses were significantly faster, χ^2^(1) = 11.90, *p* < .001, for trained items (*M* = 1151.65, *SD* = 723.16) than for foils (*M* = 1371.27, *SD* = 788.13).

##### Diverse vs non-diverse Items

We investigated whether experiencing words in diverse contexts in the learning phase led to higher rates of accuracy and faster RTs in the old/new task compared to non-diverse contexts. Only responses to the trained pseudoword stimuli were analysed. Two additional participants performed at floor (0/8 correct) meaning that the RT analysis was based on 234 participants. The random intercepts only model was used for both analyses. This model contained diversity (diverse vs. non-diverse) as a fixed effect of experimental interest. Fixed effects of Experiment (1 vs. 2) and the Experiment by diversity interaction were also included as control factors.

Contrary to our hypothesis, there was no effect of contextual diversity on accuracy, χ^2^(1) =0.26, *p* = .610, or RTs, χ^2^(1) = 1.04, *p* = .308. Mean accuracy and RTs in the two diversity conditions are summarised in Figure 2. There were no additional effects of Experiment and no Experiment by diversity interaction in either the accuracy or RT analyses.

#### Cloze task

We next investigated whether learning words in diverse contexts led to better generalisation of learned word meanings to new contexts. The random effects structure for the model used for analysis of the cloze task data consisted of random intercepts for participants and items, and a random slope for diversity by participants. The model contained diversity (diverse vs. non-diverse), cloze type (old vs. new), and the diversity by cloze type interaction as fixed effects of experimental interest. Fixed effects of Experiment, the diversity by Experiment interaction, the cloze type by Experiment interaction, and the three-way interaction between cloze type, diversity and Experiment were added into the model as additional control factors.

Overall accuracy was above chance (5 or more correct as identified using a binomial test), with participants making an average of 9.35/16 correct responses (*SD* = 4.07). This indicates that they had successfully gained some knowledge of the meanings of the pseudowords during the learning phase. Mean accuracy in each condition on the cloze task is shown in Figure 3.

There was a main effect of diversity, χ^2^(1) = 6.05, *p* = .014, with accuracy being higher for items learned in non-diverse (*M* = 4.85, *SD* = 2.41) than diverse (*M* = 4.51, *SD* = 2.41) contexts. There was also a significant interaction between cloze type and diversity, χ^2^(1) = 58.19, *p* < .001. We explored this interaction by carrying out simple effects analyses to examine the effect of diversity within each cloze type (old and new). The full model for each cloze type retained the random effects structure of the model used for the main analysis, along with the fixed effects of diversity, Experiment, and the Experiment by diversity interaction. Significance of the simple effect of diversity was determined using likelihood ratio tests in the same way as for the main analysis, using a Bonferroni-corrected significance level of .025. The effect of diversity was significant within both levels of cloze type. For cloze sentences drawn from old, familiar contexts, accuracy was significantly higher, χ^2^(1) = 45.98, *p* < .001, for words learned in non-diverse (*M* = 2.72, *SD* = 1.28) than diverse (*M* = 2.13, *SD* = 1.34) contexts. However, for cloze sentences drawn from new unfamiliar contexts, accuracy was significantly higher, χ^2^(1) = 8.39, *p* = .004, for words learned in diverse (*M* = 2.38, *SD* = 1.33) than non-diverse (*M* = 2.13, *SD* = 1.35) contexts. The results were therefore in line with our hypothesis.

There were also significant interactions between diversity and Experiment, cloze type and Experiment, and a three-way interaction between diversity, cloze type, and Experiment. The full report of the interactions can be accessed at https://osf.io/dx36f/. Importantly, the diversity by cloze type interaction was significant in both Experiment 1, χ^2^(1) = 28.94, *p* < .001, and Experiment 2, χ^2^(1) = 34.31, *p* < .001, and followed the same pattern as the main analysis. That is, in both experiments accuracy was higher for cloze sentences drawn from old contexts for words learned in non-diverse than diverse contexts, whereas for sentences drawn from new contexts accuracy was higher for words learned in diverse than non-diverse contexts.

## Discussion

This study examined whether variations in contextual diversity affect the learning of new word forms and meanings through reading. Overall, we found that contextual diversity did not significantly impact learning of word forms as measured by an old/new task that assessed orthographic processing. In contrast, contextual diversity did significantly influence learning of word meanings as measured by a cloze task that assessed semantic processing, in which participants had to select the correct newly learned word to complete a sentence. Specifically, learning new words in non-diverse relative to diverse contexts led to better use of their meanings in familiar contexts, whereas learning new words in diverse relative to non-diverse contexts led to better generalisation of their meanings to new contexts.

In the old/new task, participants learned the new word forms, as demonstrated by overall high accuracy on this task. However, we did not observe any effect of contextual diversity on word form learning. This is contrary to our first hypothesis, which predicted a diversity advantage for both RT and accuracy. It should be noted that this diversity advantage has not been consistently demonstrated in similar studies. Bolger et al. (2008) found no effect of diversity on word form learning, whereas Johns et al. (2016) reported an accuracy and RT advantage for words learned in diverse contexts. In Experiment 1, Mak et al. (2021) found an accuracy advantage for words learned in non-diverse contexts but no effect of diversity on RTs. However, in their second experiment, that provided participants with an “anchoring opportunity” (repeatedly experiencing words in one context before diversity was introduced), word recognition was faster and more accurate for words learned in diverse contexts.

There are a number of potential factors which differ between these previous studies as well as the present study. These include whether word learning was explicit or incidental, how many words were learned, whether they were presented in paragraphs or sentences, and the number of exposures to each word. However, word recognition accuracy across all of these studies was greater than 80%, therefore differences in learning may not account for the discrepant findings. Instead, perhaps, it is differences in the tasks used to assess word form knowledge that is the source of these inconsistencies. In our task and that used by Bolger et al. (2008), pseudoword foils were orthographically similar to the targets and response latencies were relatively long, whereas both Mak et al. (2021) and Johns et al. (2016) used pseudoword foils that were not closely matched to targets, and response latencies were comparably shorter. It may therefore be that effects of contextual diversity differ depending on whether word form identification can be based on overall familiarity or requires precise orthographic discrimination (Armstrong & Plaut, 2016; Hino & Lupker, 1996). However, it is unclear how a change in the relative reliance on semantic versus orthographic information would influence the direction of a diversity effect. Further research is needed to fully understand how orthographic and semantic knowledge interact in tasks designed to assess word recognition.

It is also possible that repeating the target words 10 times could have caused the difference between the two conditions to diminish. However, as other studies using comparable paradigms have found a reliable difference in diversity conditions using a similar number of repetitions (Johns et al., 2016; Mak et al., 2021) this is unlikely to account for the null effect. Nevertheless, it is possible that the nature of the learning materials used may have played a role in the observed null effect. In both Johns et al. and Mak et al., participants learned pseudowords by reading complex paragraphs whereas our participants learned the new words through reading short sentences. It could be that participants were better able to focus on learning the word forms than in these studies where more resources may have been allocated to text comprehension.

A more general concern lies with the nature of the old/new task itself. It is difficult to achieve appropriate power using lexical decision type tasks in learning studies as only a limited number of new words can be taught within a single training session. Our study is no exception to this. For a repeated-measures design Brysbaert and Stevens (2018) recommend 1,600 as a minimum of observations per condition, and although our sample size was considerably larger than those of similar learning studies, due to the small number of items learned we were unable to achieve this. Johns et al. (2016) and Mak et al. (2021) attempted to overcome this issue by repeating their pseudoword and foil stimuli five and three times respectively, whereas we presented ours only once. However, repeating the trained stimuli may have affected how easily they were recognised on subsequent trials. Future work should therefore consider using alternative tasks to assess form learning.

The cloze task showed clear effects of contextual diversity on the learning of new word meanings. Words learned in non-diverse relative to diverse contexts were better applied in familiar contexts, whereas words learned in diverse relative to non-diverse contexts were better generalised to new contexts. This supports our second hypothesis and, in line with the lexical legacy hypothesis (Nation, 2017), suggests that the degree of overlap between the contexts in which a word is experienced has consequences for developing semantic representations. Specifically, our results support the idea that experiencing words in diverse contexts may promote formation of a more flexible, decontextualised meaning representation that is then easier to generalise to new contexts. On the other hand, non-diverse contexts may favour extraction of a stable word meaning representation that is reinforced over subsequent encounters. This results in a more context-bound meaning representation that is easily used in the same context, but is difficult to generalise to a new context. Eye-tracking evidence also suggests that words experienced in contextually diverse, rather than repeated, contexts are better identified and integrated into new contexts (Pagán & Nation, 2019). Our study extends these findings by demonstrating that when low diversity is operationalised using a more naturalistic, graded measure rather than pure repetition, high diversity still leads to an advantage when learned words must be used in new contexts. Furthermore, our study is the first to include an offline semantic post-test to explicitly test generalisation of knowledge. It therefore fills an important gap in the literature, demonstrating that repeated encounters with a word in diverse contexts allows adults to extract enough semantic information not only to *recognise* that word in a new context, but also to explicitly generalise about its meaning. It is important that future studies do not only use semantic outcome measures that favour words learned in non-diverse environments (e.g., synonym judgement, definition matching), but instead include a task like the one used here that tests generalisation of meanings to novel contexts. Future research should also seek to combine implicit measures, such as eye-tracking, with offline behavioural tests to assess how contextual diversity affects the accumulation of word knowledge over encounters.

It is worth noting that our design largely replicated Joseph and Nation (2018) who did not find an effect of contextual diversity on the cloze task. The most significant difference is that their study was conducted with children and ours was conducted with adults. However, there are some other methodological differences worth mentioning. Our participants would likely have been familiar with the original target words and, as such, were perhaps learning a new form-to-existing-meaning mapping rather than a new meaning along with a new form. It has been demonstrated that learning new meanings is easier when a person already has some pre-existing knowledge of that concept (Havas et al., 2018). It may be that this allowed our participants to extract the core meanings of the new words in fewer exposures, which in turn allowed them to benefit from the diversity of these exposures. It may also explain why we obtained an effect of diversity without providing our participants with an anchoring opportunity. Although Mak et al.’s (2021) target pseudowords also replaced real English words, these were very low frequency and participants were unlikely to have had any prior familiarity with the word/underlying concept.

### Future directions

Contextual diversity has been defined and operationalised inconsistently across different word learning studies. While some have defined low diversity as contexts drawn from a single discourse topic (Johns et al., 2016; Joseph & Nation, 2018; Mak et al., 2021), others have used repetition of the exact same material (Bolger et al., 2008; Pagán & Nation, 2019). What constitutes high diversity is also unclear. For instance, in our experiment (and Joseph and Nation’s original study) sentences in the high diversity condition were drawn from 10 discourse topics. In comparison, Johns et al. used five topics and Mak et al. used six in Experiment 1, but only two in Experiment 2. Does high contextual diversity simply mean experiencing a word in more than one context, or is there a minimum number of contexts needed for a diversity advantage to emerge? Future research should aim to address these questions.

Another issue is the contrast between the mixed results from word learning studies and those from corpus studies, which consistently report that diversity facilitates lexical decision, but impairs semantic judgements (Hoffman & Woollams, 2015; Jones et al., 2012). One disadvantage of using a word learning paradigm is the limited number of exposures participants are typically given. For example, in our study and Mak et al. (2021, Experiment 1) participants saw each target pseudoword 10 times, participants in Johns et al. (2016) were given five exposures, and those in Mak et al. (Experiment 2) received 12 exposures. Stimuli used in corpus studies would likely have been experienced far more often with exposures distributed over the participant’s lifetime, rather than within a single experimental session. Future research should seek to establish the number of exposures needed for an effect of diversity to emerge and investigate the long-term effects of contextual diversity on new word learning by spacing learning over multiple sessions. Studies should also consider potential effects of overnight sleep, which may differ for words learned in high versus low diversity conditions (James et al., 2020).

There is also the possibility that corpus derived measures of contextual diversity and contextual diversity as induced in learning studies may not tap into the same underlying construct. Within corpus studies, contexts are defined as distinct documents (Jones et al., 2012) or sections of text (Hoffman et al., 2013; Hoffman & Woollams, 2015) without the content of these initially being taken into account. Measures of diversity are then retrospectively computed as the average similarity across all the contexts in which a word occurs, giving a measure of the degree to which the contexts in which a word is used are linguistically distinct. This approach makes it difficult to disentangle effects of contextual diversity from those of polysemy—the number of semantically related “senses” that a word has. For example, *twist* has several definitions including to make into a coil or spiral, to operate by turning, and to alter the shape of (Rodd et al., 2004). Previous work has shown that polysemous words enjoy a processing advantage in lexical decision tasks but show a disadvantage in tasks of semantic classification (Hino & Lupker, 1996; Rodd, 2004; Rodd et al., 2002; Yap et al., 2011; for a review see Rodd, 2020). It is plausible that polysemy and contextual diversity have been confounded within the two main diversity metrics derived from corpus studies, namely, semantic distinctiveness and semantic diversity. Jones et al. (2012) did not control for polysemy when validating their semantic distinctiveness measure, and the original Hoffman et al. (2013) metric was explicitly intended to capture semantic ambiguity rather than contextual variation.

On the other hand, learning studies have typically defined contexts as varying topics. Operationalising contextual diversity in this way could provide an opportunity to separate potential effects of polysemy and contextual diversity on word learning. However, this has not been explored in the existing literature. Neither Mak et al. (2021) nor Johns et al. (2016) controlled for polysemy, using a mixture of words with only a single sense as defined in WordNet (Miller, 1995), for example *avidity* (Mak et al.), and polysemous words, for example *constellation* (Johns et al.; Mak et al.). The training materials created then emphasised different senses of the word, for example constellation could refer to a clustering of symptoms of a disease or the arrangement of stars in the sky (Rosa et al., 2017). Although Joseph and Nation (2018) also used a mixture of monosemous and polysemous words, when a polysemous word was used, sentence stimuli consistently emphasised only one of the word’s senses in both the diverse and non-diverse conditions (e.g., *accumulate* always meant to collect or gather). Differing degrees of polysemy among base words could possibly underlie some of the inconsistencies in the results of learning studies to date and should be taken into consideration in future research. LSA may also not be an appropriate metric for assessing the validity of a diversity manipulation, since there is some controversy over the extent to which it accurately captures semantic variation in the documents in which a word occurs (Cevoli et al., 2021; Li & Joanisse, 2021). Joseph and Nation did not use LSA to evaluate the similarity of their training sentences. Instead, a separate group of adults read all 10 sentences for each word and rated how similar they were in topic. Considering the potential limitations of LSA, this may be a more appropriate way of assessing topic overlap.

## Conclusion

Our study fills an important gap in the literature by confirming a key prediction of the lexical legacy and lexical quality hypotheses; that learning words in contextually diverse environments leads to extraction of a meaning that is more generalisable and less bound by context. In addition, we also demonstrated that experiencing words in non-diverse contexts benefits participants when they must use them in a familiar context, indicating that differing degrees of contextual diversity have different consequences for developing semantic representations. We have also highlighted some important points that need to be addressed moving forward. At present, the lack of consistency across learning studies makes it difficult to compare results and draw firm conclusions as to the relative benefits of contextual diversity for form and meaning learning. Future studies should seek to standardise what constitutes high and low diversity, investigate how diversity affects word learning over time, and include an outcome measure that explicitly tests generalisation of word meanings to new contexts. Word learning studies are key to disentangling these issues, as they permit researchers to vary these factors while controlling for other linguistic variables, such as polysemy.

## Notes

1. Note that this differs from the total number of participants reported in the analysis plan due to differences in the exclusion criteria applied to the data in the student projects and in this study.

2. Joseph and Nation designed sentence stimuli for “divulged” and “thwarted,” but these were not used in their original experiment as divulged had orthographic overlap with other candidate target words and thwarted contained a rare initial trigram. As we replaced these words with pseudowords, these issues did not affect our study.

3. We did not include random slopes for Experiment or any of the interactions with Experiment as this control factor was not of experimental interest.
